# Validation of analytical methods in compliance with good manufacturing practice: a practical approach

**DOI:** 10.1186/1479-5876-11-197

**Published:** 2013-08-27

**Authors:** Deborah Rustichelli, Sara Castiglia, Monica Gunetti, Katia Mareschi, Elena Signorino, Michela Muraro, Laura Castello, Fiorella Sanavio, Marco Leone, Ivana Ferrero, Franca Fagioli

**Affiliations:** 1 Paediatric Onco-Hematology, Stem Cell Transplantation and Cellular Therapy Division, City of Science and Health of Turin, Regina Margherita Children’s Hospital, P.zza Polonia 94, Turin 10126, Italy; 2 Department of Public Health and Paediatrics, University of Turin, P.zza Polonia 94, 10126, Turin, Italy

**Keywords:** Cell therapy, Quality control, Stem cell

## Abstract

**Background:**

The quality and safety of cell therapy products must be maintained throughout their production and quality control cycle, ensuring their final use in the patient. We validated the Lymulus Amebocyte Lysate (LAL) test and immunophenotype according to International Conference on Harmonization Q2 Guidelines and the EU Pharmacopoeia, considering accuracy, precision, repeatability, linearity and range.

**Methods:**

For the endotoxin test we used a kinetic chromogenic LAL test. As this is a limit test for the control of impurities, in compliance with International Conference on Harmonization Q2 Guidelines and the EU Pharmacopoeia, we evaluated the specificity and detection limit.

For the immunophenotype test, an identity test, we evaluated specificity through the *Fluorescence Minus One* method and we repeated all experiments thrice to verify precision. The immunophenotype validation required a performance qualification of the flow cytometer using two types of standard beads which have to be used daily to check cytometer reproducibly set up. The results were compared together.

Collected data were statistically analyzed calculating mean, standard deviation and coefficient of variation percentage (CV%).

**Results:**

The LAL test is repeatable and specific. The spike recovery value of each sample was between 0.25 EU/ml and 1 EU/ml with a CV% < 10%. The correlation coefficient (≥ 0.980) and CV% (< 10%) of the standard curve tested in duplicate showed the test's linearity and a minimum detectable concentration value of 0.005 EU/ml.

The immunophenotype method performed thrice on our cell therapy products is specific and repeatable as showed by CV% *inter* -experiment < 10%.

**Conclusions:**

Our data demonstrated that validated analytical procedures are suitable as quality controls for the batch release of cell therapy products.

Our paper could offer an important contribution for the scientific community in the field of CTPs, above all to small Cell Factories such as ours, where it is not always possible to have CFR21 compliant software.

## Background

The success of advanced therapy-based approaches is highly dependent upon the development of standardized protocols according to Good Manufacturing Practice (GMP) [1], including production and quality control processes.

The quality and safety of cell therapy products (CTP) must be maintained throughout their production and quality control (QC) cycle, ensuring their final use in the patient. According to International Conference on Harmonization Q2 (ICH Q2) Guidelines [2] and the European (EU) Pharmacopoeia [3], the QC process should be validated to confirm that the analytical procedure employed for a specific test is suitable for its intended use. Results from method validation can be used to judge the quality, reliability and consistency of analytical results.

The four most common types of analytical methods, each with its own set of validation requirements, are identity tests, quantitative tests for impurity content, limit tests for the control of impurities, potency tests.

The validity of an analytical method should be demonstrated using samples or standards that are similar to routinely analyzed unknown samples. The process should follow a validation protocol, also considering instruments, supplies and reagents.

The validation strategy described in the validation protocol should clearly define the roles and responsibilities of each step involved in the validation of analytical methods.

The elements of the analytical method requiring proof through validation as contained in the ICH Q2A guidelines are specificity, accuracy, precision, repeatability, linearity and range [2,4].

In this work, we report the validation processes of a immunophenotype method as an identity test and Lymulus Amebocyte Lysate (LAL) test as a limit test for the control of impurities, as a conclusion of a validation process that also including a potency test, as previously reported [5].

The LAL test is used to assess that CTPs given to patients are negative for bacterial endotoxin, that is the lipopolysaccharide (LPS) component of the cell wall of Gram-negative bacteria. The pathological effects of endotoxin, when injected, are a rapid increase in core body temperature followed by extremely rapid and severe shock, often followed by death before the cause is even diagnosed.

The principle aim of this assay is a reaction between LPS and a lysate contained in amoebocyte cells derived from the blood of Limulus Polyphemus [6]. The LAL in presence of bacterial endotoxins activate an enzymatic reaction that leads to a local blood coagulation cascade.

The immunophenotype analysis is a multiparametric technique to identify cell subpopulations. Cells can be identified on the bases of their size and by using fluorescent monoclonal antibodies that bind to intra-cellular and surface antigens. For CTPs, cell identity is a fundamental parameter to be assessed in GMP quality controls [7].

Using well-designed experiment and statistically relevant analysis, method validation can be accomplished in accordance with ICH guidelines [2]. Thus, to perform test validation, we assessed a detailed validation protocol for each test. For our study, we chose three cell populations and respective supernatants: bone marrow mesenchymal stem cells (BM MSCs) and Cytotoxic T Lymphocytes (CTLs), both cell therapy products that we will produce, in GMP conditions, for clinical trials of immunotherapy and regenerative medicine, and dendritic cells (DCs) used as antigen presenting cells (APCs) to generate CTLs.

## Materials and methods

### Cell source

#### *BM MSCs isolation and expansion*

BM MSCs were isolated from humans obtained by aspiration from the posterior iliac crest of healthy donors after written informed consent. The frequency BM MSC was about 1/10^4^ cells [8]. Briefly, whole bone marrow (wBM) was seeded at a density of 100,000/cm^2^ in Mesenchymal Stem Cell Growth Medium (MesenCult® Proliferation Kit; Human, Stemcell technologies, Vancouver, BC, Canada) containing 10% of fetal bovine serum (FBS) in 75 or 150 cm^2^ T-flasks and maintained at 37°C with an atmosphere of 5% CO_2_. After 5 days, the non-adherent cells were removed and re-feed every 3–4 days; at confluence, they were detached, and re-plated at different densities for one to four passages [9].

To perform immunophenotype analysis BM MSCs, at the end of culture when confluent, were detached, washed with Phosphate Buffered Saline (PBS) 1X (200 g for 10 minutes) and resuspended in PBS 1X. BM MSCs and supernatant at different dilutions were tested for endotoxins.

#### *PBMCs isolation*

Peripheral blood mononuclear cells (PBMCs) were prepared from buffy coats obtained from healthy donors kindly provided by the local blood bank after informed consent. PBMCs were layered on Hystopaque (Sigma Aldrich, Milan, Italy) gradient (1.077 g/ml density). The cells were centrifuged at 400 g for 30 minutes. The cells in the interphase were collected and washed twice with PBS 1X (200 g for 10 minutes).

#### *Dendritic cells (DCs) generation*

Dendritic cells (DCs) were generated from PBMCs after adhesion for two hours at 37°C with an atmosphere of 5% CO_2._ After two hours, the non-adherent cells were removed and the adherent cells were cultured in CellGro DC medium (CellGenix, Freiburg, Germany) supplemented with recombinant human (rh) granulocyte-macrophage colony-stimulating factor (rhGM-CSF; CellGenix) and rh Interleukin (IL)-4 (CellGenix) [10-12]. Fresh cytokines were added on day 3. On day 5, adherent cells were maturated with a cytochine cocktail for 48 hours: rhGM-CSF, rhIL-4, rh-IL-6 (CellGenix), rhIL-1β (CellGenix), rh Tumor Necrosis Factor-alpha (TNF-α; Cell Genix), rh Prostaglandin E2 (PGE2; Cayman Chemical, Ann Arbor, MI, USA).

On day 5 and on day 7, immature DCs (iDCs) and mature DCs (mDCs) were immunophenotyped respectively.

#### *Cytotoxic T Lymphocyte (CTLs) generation and expansion*

PBMCs cells obtained were co-cultured with mDCs loaded with irradiated human osteosarcoma cell lines SJSA1 derived from American Type Culture Collection (ATCC, Rockville, MD, USA,) (ratio 1:10) in CellGro Serum-Free Stem Cell Growth medium (SCGM; CellGenix) with 5% Human Serum (HS; Lonza, Verviers, Belgium) supplemented with recombinant human Interleukin-7 (rhIL-7; CellGenix), recombinant human Interleukin-12 (rhIL-12; CellGenix) and recombinant human Interleukin-15 (rhIL-15; CellGenix) for seven days.

On day 7 the CTLs were re-stimulated with fresh DCs obtained after PBMC adhesion, as explained above, loaded with irradiated human osteosarcoma cell lines in SCGM Medium with 5% HS supplemented with rhIL-2 and rhIL-15 for seven days.

CTLs stimulated cells were expanded in an antigen independent manner [13] by co-culture with irradiated autologous PBMCs in SCGM with 5% HS supplemented with rhIL-2 and Muromonab-CD3 (OKT3; Milteny, Bergisch Gladbach, Germany) for 7 days. After expansion CTLs immunophenotyping was performed after washing with PBS 1X (200 g for 10 minutes).

CTLs and supernatant at different dilutions were tested for endotoxins.

### Endotoxin test

LAL assay is a quantitative method to detect Gram - derived endotoxin in a solution. LAL is an aqueous extract of blood cells (amebocytes) from the "horseshoe crab", Limulus Polyphemus. The endotoxin catalyzes the activation of a proenzyme in the LAL. The rate of reaction depends on the concentration of endotoxin present. The activated enzyme is able to break the p-NitroAniline (pNA) bond with the colorless artificial substrate. The pNA released produces a yellow element quantitatively photometrically determinable at 405 nm. The time required before the appearance of a yellow color (reaction time) is inversely proportional to the amount of endotoxin present. The concentration of endotoxin in a sample is calculated from its reaction time compared to the reaction time of solutions containing known amounts of endotoxin standard.

To detect the Gram - bacterial endotoxin on our CTPs, we used the LAL Kinetic-K-QLC kit (Lonza). Standard curve with 0.005 endotoxin unit EU/mL was used in this assay. The high and low points in a valid standard curve determine the lower and upper levels of endotoxin that can be detected. The correlation coefficient (CC) of the calculated standard curve should be ≥ 0.980. The assay was assessed on 100 μL supernatant by incubating the samples and the calibrators at 37°C in the presence of the LAL for 1 hour and 40 minutes in a microplate reader ELX −808 (Lonza).

The endotoxin test is a limit test for the control of impurities, in compliance with ICHQ2 guidelines [2] and the EU Pharmacopoeia [3], so, we evaluated specificity and detection limit.

**Figure 1:**
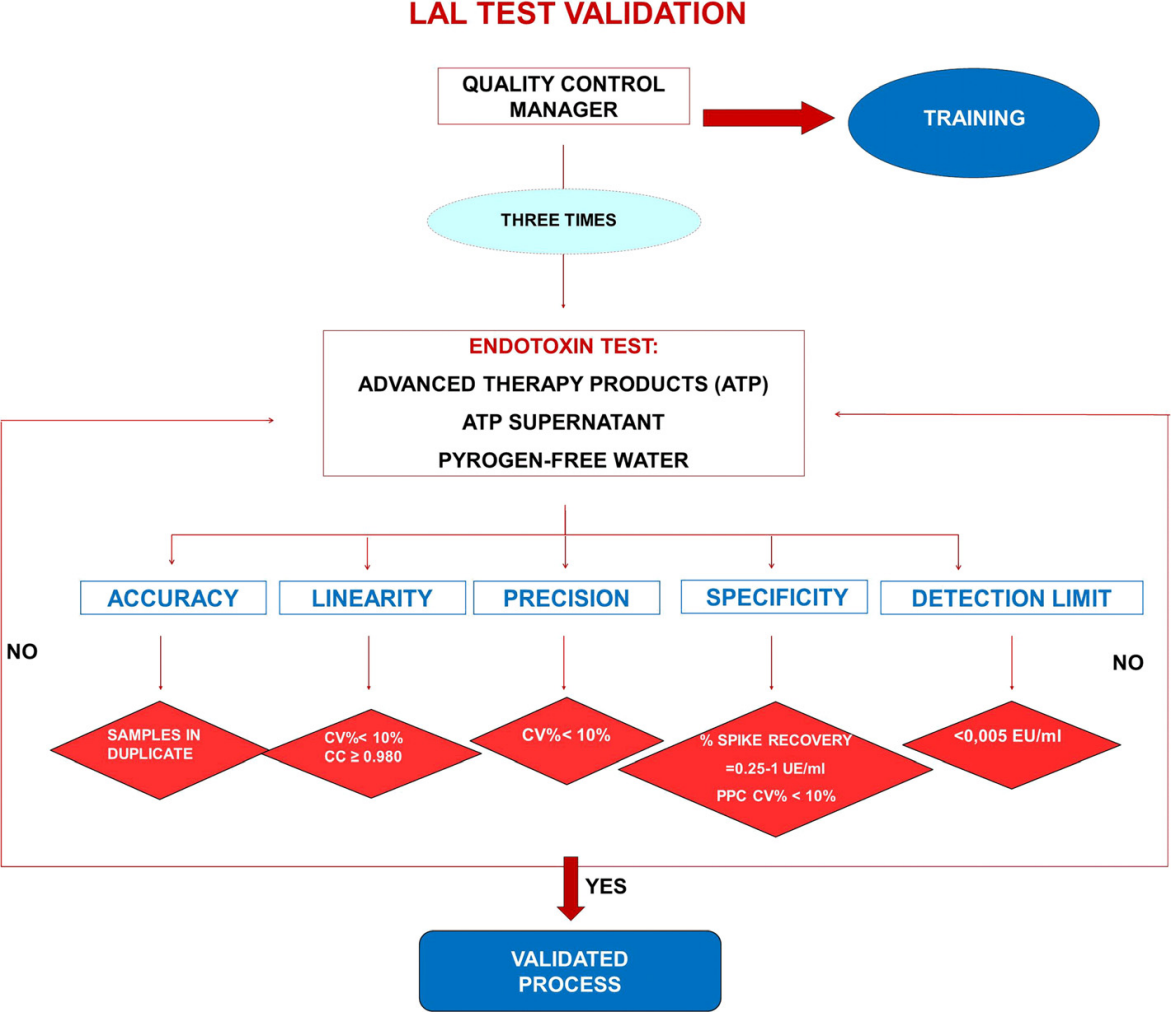
**LAL test validation protocol flow-chart.** The test was performed three times under the same operating conditions by the QC manager on the same samples (CTPs, CTPs supernatant, pyrogen-free water) to test precision. According to ICH Q2 we evaluated specificity and the detection limit. To evaluate accuracy, the assay includes seeding each sample in duplicate. For linearity, a standard curve with 0.005 endotoxin unit EU/mL was used. The acceptance criteria were: spike recovery between 0.25 EU/ml – 1 EU/ml with a CV% < 10, standard curve with CV < 10% and correlation coefficient ≥ 0.980.

The endotoxin test validation protocol was performed as shown in the flow chart (Figure 1).

The test was performed on supernatant at different dilutions, on CTPs at different concentrations, and on pyrogen-free water as negative control. For this analysis we tested the supernatants containing FBS and HS, added as explain above, to BM MSCs and CTLs culture medium and those composed of saline (FS) and albumin as a medium for the infusion of cell therapy products in the patient. The CTP’s supernatant was diluted in LAL Reagent Water (Lonza) considering the maximum valid dilution (MVD) equal to 100. To exclude the possibility of false negatives, we validated the freezing of the supernatant by running the test on the supernatant fresh and thawed. We also performed the test on the supernatant heated to 75°C to exclude the effect of trypsin, which can give interference (Table 1). All the tubes, water and pipette-tips were certified pyrogen-free.

**Table 1:** LAL test: list of samples analyzed

**Sample number**	**Samples**
**1**	PYROGEN-FREE WATER
**2**	SN + 10%FBS 1:10
**3**	SN + 10%FBS 1:50
**4**	SN + 10%FBS 1:80
**5**	SN + 10%FBS THAWED 1: 10
**6**	SN + 10%FBS THAWED 1:50
**7**	SN + 10%FBS THAWED 1:80
**8**	SN + 10%FBS HEATED 1:10
**9**	SN + 10%FBS HEATED 1:50
**10**	SN + 10%FBS HEATED 1:80
**11**	FS + 5%ALBUMIN 1:10
**12**	FS + 5%ALBUMIN 1:50
**13**	FS + 5%ALBUMIN 1:80
**14**	BM MSCs 1X10^3^ cells/ml
**15**	BM MSCs 1X10^2^ cells/ml
**16**	CTLs 1X10^3^ cells/ml
**17**	CTLs 1X10^2^ cells/ml
**18**	SN + 5%HS 1:10
**19**	SN + 5%HS 1:50
**20**	SN + 5%HS 1:80
**21**	SN + 5%HS THAWED 1:10
**22**	SN + 5%HS THAWED 1:50
**23**	SN + 5%HS THAWED 1:80

The test was performed on supernatant at different dilutions, on CTPs at different concentrations, and on pyrogen-free water as negative control.

*Abbreviations:SN* supernatant, *FBS* fetal bovine serum, *FS* saline, *HS* human serum.

To verify precision, the LAL test was performed three times under the same operating conditions by quality control (QC) manager on the same samples.

To evaluate assay accuracy, the test includes seeding each sample in duplicate.

Each sample must be accompanied by a positive product control (PPC) that is a sample of product to which a known amount of endotoxin (0.5 EU/ml) has been added. To verify test specificity, that is the ability to detect the analyte in the presence of interfering substances, we evaluated the spike recovery (the amount of endotoxin recovered) for each sample.

### *Immunophenotyping analysis*

**Figure 2:**
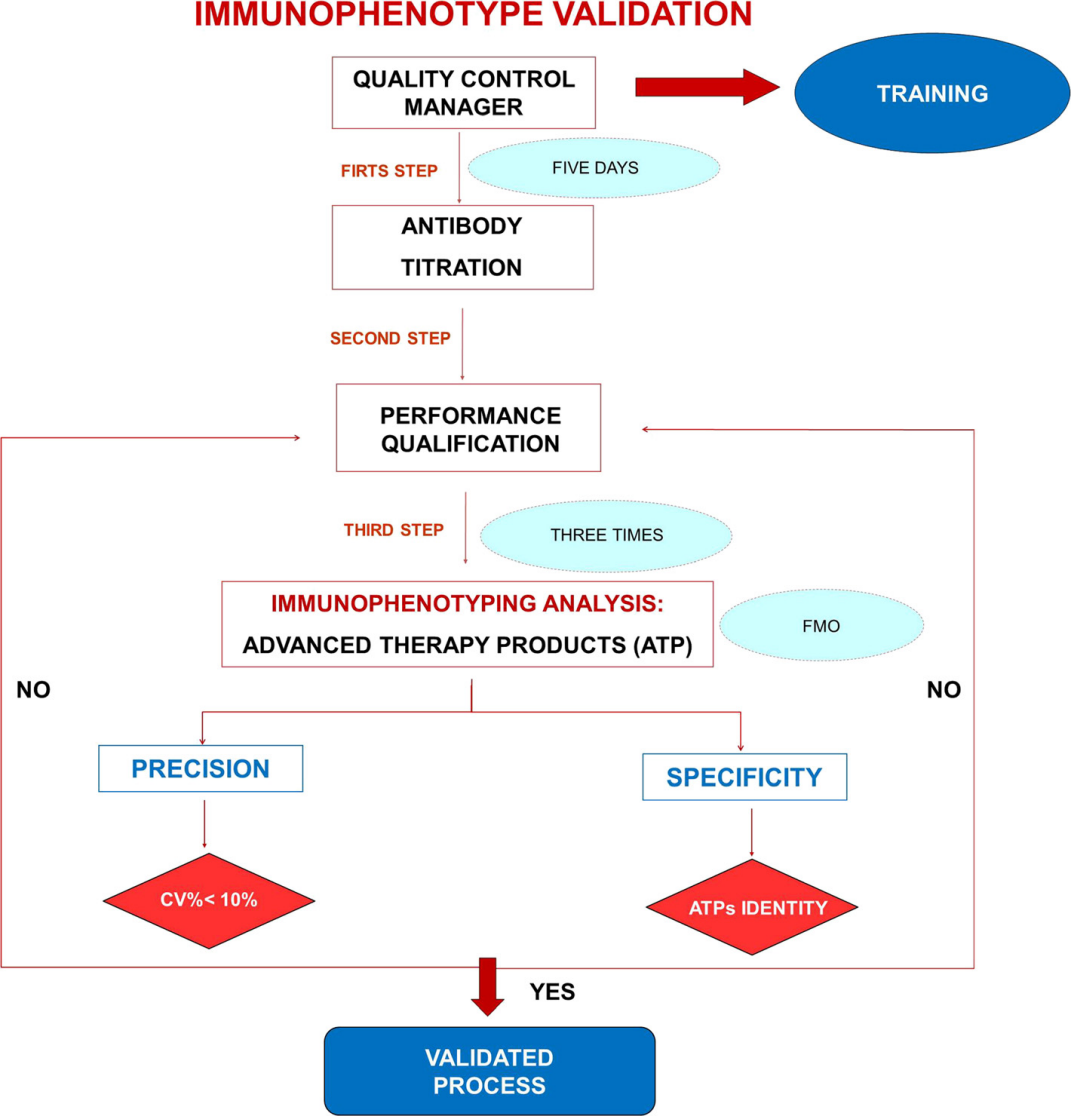
**Immunophenotype validation protocol flow-chart.** The immunophenotype validation protocol required: a first step which is the titration of each antibody performed by using scalar antibody dilution; a second step, named Performance Qualification (PQ), during which the QC manager used two types of standard beads to check cytometer reproducibly over time. Immunophenotyping analysis is an identity test to evaluate specificity by using FMO method. The test was performed three times to test precision. The acceptance criteria were: inter-experiment CV%  ≤ 10%, BM MSCs positive for CD90, CD73, CD105 and negative for CD45, CD14, CD34, CD19 and HLADR; mDCs positive for CD80, CD86, CD83, CD40, CD11c and HLADR at a high level; CTLs positive for CD3+, CD3 + CD4+, CD3 + CD8+, CD56 + CD3- at a low level and negative for CD19.

The immunophenotype validation protocol (Figure 2) required a first step which is the titration of each antibody performed by using scalar antibody dilution. The better antibody concentration was that with higher resolution index, that is a greater separation between the negative control peaks and the labeled samples.

A second step was Performance Qualification (PQ), in compliance with ICHQ2 [2], that demonstrates that the process or equipment performs as intended in a consistent manner over time. The resolution index was calculated as follows: IR = X_i_-X_0_/√ SD_i_^2^ + SD_0_^2^ where X_i_ is the mean fluorescence intensity (MFI) of the positive cell population, X_0_ is the mean fluorescence intensity of the negative cell population, SDi is the MFI standard deviation of the positive cell population and SD_0_ is the MFI standard deviation of the negative cell population. We carried out titration for the following antibodies (mAb) combined in different panels as described below: CD45- fluorescein isothiocyanate (FITC), CD34-FITC, CD14-FITC, HLADR-phycoerythrin (PE), CD19–allophycocyanin (APC), CD90-FITC, CD73-PE, CD83-FITC, CD40-PE, CD80-FITC, CD86-PE, CD11c-APC, HLADR- peridinin-chlorophyll protein cyanine 5.5 (PerCP-Cy5.5), CD45RA FITC/CD45RO PE/CD3 PercP/CD8 APC, CD45RA FITC/CD45RO PE/CD3 PercP/CD4 APC, CD3 FITC/CD8 PE/CD4 APC/CD45 PercP, CD3 FITC/CD56-16 PE/CD19 APC/CD45 PercP (Becton Dickinson), CD105-APC (Miltenyi Biotech).

To perform PQ, the QC Manager, over five consecutive days used BD FACS 7-Color Setup Beads (Becton Dickinson, San Jose, CA, USA) and CS&T beads (Becton Dickinson), two types of standard beads which have to be used daily to check cytometer reproducibly set up. We checked our Levey Jennings graph of each type of bead in order to evaluate time trend. The results obtained from both beads were compared together.

As the immunophenotype analysis, in compliance with ICHQ2 [2] guidelines and the EU Pharmacopoeia [3], is an identity test, we evaluated specificity.

We tested specificity on BM MSCs, iDCs, mDCs and CTLs, by using Fluorescence Minus One method (FMO): each cell population was stained with all the reagents, except one, at a time, in order to verify whether in the absence of one antibody, the labeled cells were negative for the removed one.

BM MSCs were labeled with the following mAb panels : anti-human CD45–CD34-CD14-FITC/ HLADR-PE/ CD19–APC, CD90-FITC/ CD73-PE/ CD105-APC.

iDC and mDC staining was performed with anti-human CD83-FITC/ CD40-PE/ HLADR PerCP-Cy5.5/ CD11c-APC, CD80-FITC/ CD86-PE HLADR- PerCP-Cy5.5, CD11c-APC.

CTLs immunophenotyping was performed with the following mAbs panels: anti human CD3 FITC/CD8 PE/CD4 APC/CD45 PercP, CD3 FITC/CD56-16 PE/CD19 APC/CD45 PercP. CTL effectors were labeled with anti-human CD45RA FITC/CD45RO PE/CD3 PercP/CD8 APC, CD45RA FITC/CD45RO PE/CD3 PercP/CD4 APC.

For each antibody panel, 500,000 cells/100 μl were stained for 20 minutes.

The labeled cells were thoroughly washed with PBS 1× (200 g for 10 minutes) and analyzed on a FACSCanto II (Becton Dickinson) with the DIVA software program. The percentage of positive cells was calculated using the FMO cells as a negative control for each antigen expression.

To test inter-experiment repeatability all immunophenotyping tests on our CTPs were repeated three times by the QC Manager.

### Data analysis and statistical approach

The endotoxin test result was considered valid when the spike recovery was between 0.25 EU/ml – 1 EU/ml with a CV% less than 10%, a standard curve with CV% less than 10% and a correlation coefficient ≥ 0.980.

To test the precision of the immunophenotype analysis, we calculated mean, SD and CV% of the Mean Fluorescence Intensity (MFI) of each marker considering the results of triplicate experiments.

The immunophenotype method was considered specific when: BM MSCs were positive (≥ 70%) for CD90, CD73, CD105 and negative (≤ 2%) for CD45, CD14, CD34, CD19 and HLADR [14]; mDCs expressed high levels express high levels of CD80, CD86, CD83, CD40, CD11c and HLADR [12], some of which were upregulated compared to iDC; CTLs expressed for CD3+ (≥ 70%), CD3 + CD4+, CD3 + CD8+ (≥ 30%), negative for CD19 and expressed low levels of CD56 + CD3- (≤ 10%) [15].

### Equipment validation

Microplates reader ELX-808 (Lonza) and flow cytometer FACS Canto II (Becton Dickinson) were properly qualified by Installation Qualification (IQ) and Operational Qualification (OQ) according to GMP guidelines [1,2].

Micropipettes used for the tests were calibrated by the manufacturer. Furthermore a new set of pipettes every year is bought, as we considered them critical instruments in risk assessment.

### Statement of ethical approval

Bone Marrow (BM) and peripheral blood (PB) were obtained from healthy donors after written informed consent in accordance with the approval of the Ethics Committees, of the Regina Margherita, S.Anna and Mauriziano hospitals, and in compliance with the Helsinki Declaration.

## Results

### Endotoxin test

As previously explained, the assay was performed on our CTPs and supernatants using a kinetic chromogenic method. The test performed three times, under the same operating conditions by the QC Manager was repeatable (Table 2). Endotoxin concentrations in all samples were less than 0.5 EU/ml as requested by the Food and Drug Administration. The endotoxin limit for all parenteral drugs is 5 EU/Kg and for those that have an intrathecal route of administration is 0.2 EU/Kg [16]. For all tests the absolute value of CC of the standard curve tested in duplicate was ≥ 0.980 and the CV% less than 10% showed the test’s linearity. The minimum detectable concentration was 0.005 EU/ml.

**Table 2:** LAL test precision

**Sample number**	**Experiment 1 (EU/ml)**	**Experiment 2 (EU/ml)**	**Experiment 3 (EU/ml)**
1	<0.0050	<0.0050	<0.0050
2	<0.0050	<0.0050	<0.0050
3	<0.2500	<0.2500	<0.2500
4	<0.4000	<0.4000	<0.4000
5	<0.0500	<0.0500	<0.0500
6	<0.2500	<0.2500	<0.2500
7	<0.4000	<0.4000	<0.4000
8	<0.0500	<0.0500	<0.0500
9	<0.2500	<0.2500	<0.2500
10	<0.4000	<0.4000	<0.4000
11	<0.0500	<0.0500	<0.0500
12	<0.2500	<0.2500	<0.2500
13	<0.4000	<0.4000	<0.4000
14	<0.0050	<0.0050	<0.0050
15	<0.0050	<0.0050	<0.0050
16	<0.0050	<0.0050	0.0627
17	<0.0050	<0.0050	<0.0050
18	0.0667	0.0558	<0.0500
19	<0.2500	<0.2500	<0.2500
20	0.4095	<0.4000	<0.4000
21	<0.0500	<0.0500	0.065
22	<0.2500	<0.2500	<0.2500
23	<0.4000	<0.4000	<0.4000

The LAL test was performed three times under the same operating conditions by the QC manager on our CTPs and supernatants. The test was repeatable. Endotoxin concentrations in all samples (EU/ml) were ≤ 0.5 EU/ml as requested by the Food and Drug Administration. Samples analysed are given in Table 1. Samples: 1 = pyrogen free water (negative control); 2–10 = supernatants with FBS; 11–13 = supernatants with albumin; 14–17 = CTPs; 18–23 = supernatants with HS.

Pyrogen-free water used as a negative control, had an endotoxin value less than the lowest standard according to the European Pharmacopeia [3].

**Figure 3:**
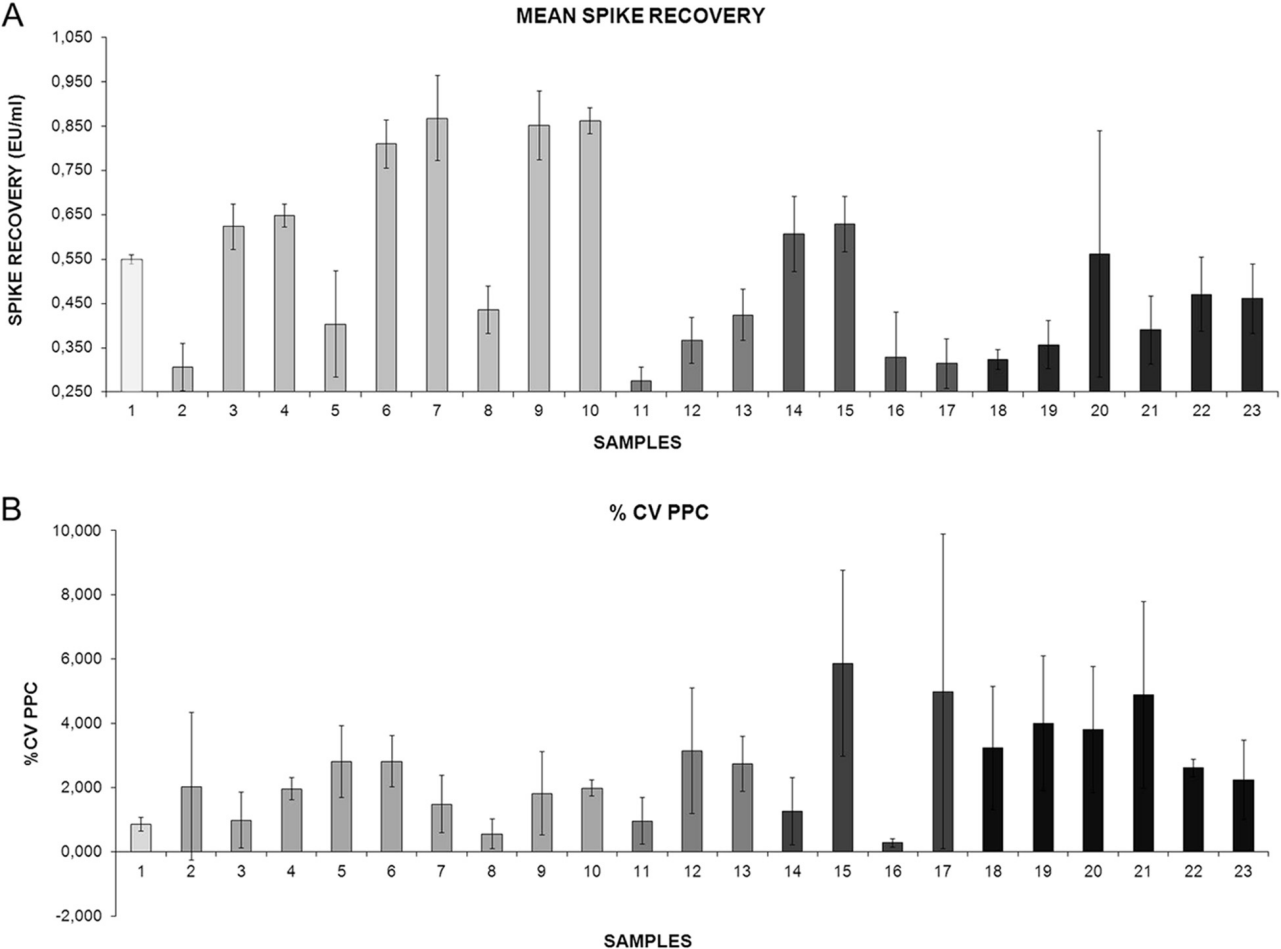
**LAL test specificity.** According to the acceptance criteria, the histogram shows that the mean spike recovery of three replicates for all samples analysed was between 0.25 EU/ml and 1EU/ml **(A)** with PPC CV% less than 10 **(B)**. Bars are SD. Samples analysed are given in Table 1. Samples: 1 = pyrogen free water (negative control); 2–10 = supernatants with FBS; 11–13 = supernatants with albumin; 14–17 = CTPs; 18–23 = supernatants with HS.

As suggested by ICHQ2 [2] we demonstrated the discrimination of the analyte in the presence of impurities by spiking all samples with known levels of endotoxin and by comparing the results obtained on un-spiked samples. According to acceptance criteria the mean spike recovery of three replicates for all samples analyzed, was between 0.25 EU/ml and 1 EU/ml with PPC CV% less than 10. These data summarized in Figure 3A and B demonstrated the test’s specificity.

#### Immunophenotyping analysis

**Figure 4:**
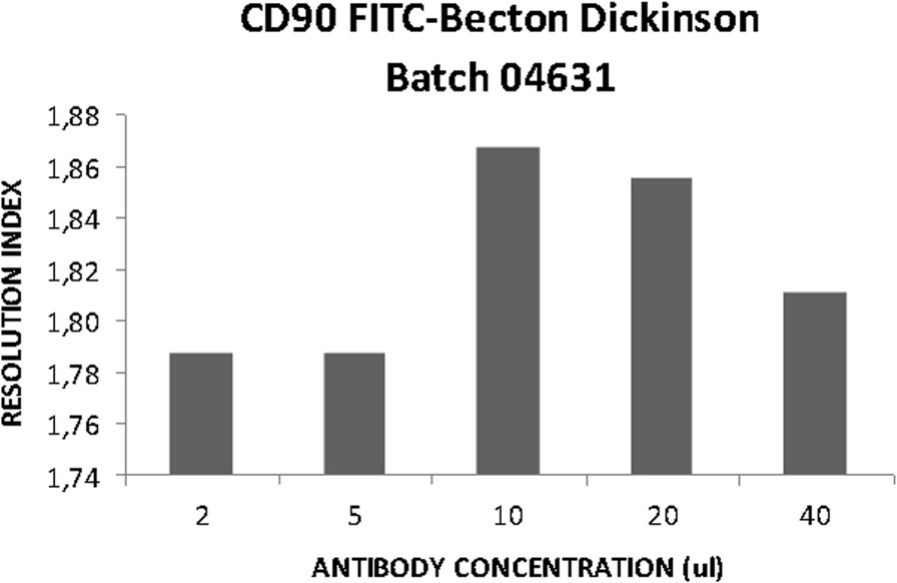
**Representative panel of antibody titration.** CD90 FITC titration on BM MSCs.

The first step of our analysis was the titration of each monoclonal antibody to be used for the immunophenotype of our CTPs. The determination of the antibody dilution constitutes the previous key step to flow cytometry analysis, since it is highly dependent on the antigen density in the cells. Ideally, each antibody concentration should be established for each sample that requires analysis [17]. To label the cell populations we chose the concentration of each antibody with the highest resolution index. The lowest antibody concentration was chosen when there was an equal resolution index (Table 3). Figure 4 is a representative panel of antibody titration.

**Table 3:** Antibody tritation

**Markers**	**Antibody concentration (μl)**				
	**5**	**10**	**20**	**40**	**80**
CD45 FITC	2.0	2.0	2.0	1.9	1.9
CD34 FITC	1.8	1.9	2.0	1.9	1.9
CD14 FITC	1.9	1.9	1.9	1.9	1.9
HLADR PE	0.8	0.7	0.7	0.8	0.9
CD73 PE	1.8	1.9	2.0	2.0	2.1
CD83 FITC	1.7	1.8	1.8	1.9	2.0
CD40 PE	1.5	1.7	1.6	1.7	1.8
CD80 FITC	1.9	1.9	1.9	1.9	2.0
CD86 PE	1.1	1.1	1.1	1.2	1.5
HLADR PercPCy5.5	1.2	1.2	1.3	1.2	1.3
**CD45RA FITC**/CD45RO PE/CD3 PercP/CD8 APC	1.7	1.9	1.5	1.5	1.7
CD45RA FITC/**CD45RO PE**/CD3 PercP/CD8 APC	2.6	3.0	2.5	2.0	2.2
CD45RA FITC/CD45RO PE/**CD3 PercP**/CD8 APC	2.5	2.5	2.5	1.9	2.1
CD45RA FITC/CD45RO PE/CD3 PercP/**CD8 APC**	2.4	3.3	2.6	2.5	1.7
**CD45RA FITC**/CD45RO PE/CD3 PercP/CD4 APC	1.8	1.7	1.4	1.3	1.4
CD45RA FITC/**CD45RO PE**/CD3 PercP/CD4 APC	2.2	2.1	2.2	2.2	2.1
CD45RA FITC/CD45RO PE/**CD3 PercP**/CD4 APC	2.5	2.4	2.4	2.2	2.1
CD45RA FITC/CD45RO PE/CD3 PercP/**CD4 APC**	5.1	5.0	4.5	4.6	4.5
**CD3 FITC**/CD8 PE/CD4 APC/CD45 PercP	2.2	2.0	2.0	2.0	1.9
CD3 FITC/**CD8 PE**/CD4 APC/CD45 PercP	2.6	1.8	1.8	1.2	1.2
CD3 FITC/CD8 PE/**CD4 APC**/CD45 PercP	4.3	4.2	4.0	4.0	4.0
CD3 FITC/CD8 PE/CD4 APC/**CD45 PercP**	4.0	3.9	3.8	3.7	3.6
**CD3 FITC**/CD56-16 PE/CD19 APC/CD45 PercP	2.1	2.4	2.4	2.3	2.1
CD3 FITC/**CD56-16 PE**/CD19 APC/CD45 PercP	0.9	1.2	1.6	1.2	1.1
CD3 FITC/CD56-16 PE/**CD19 APC**/CD45 PercP	2.8	1.8	3.4	2.4	1.9
CD3 FITC/CD56-16 PE/CD19 APC/**CD45 PercP**	3.1	3.3	3.7	3.7	3.7
**Markers**	**Antibody concentration (μl)**				
	**2**	**5**	**10**	**20**	**40**
CD105 APC	1.8	1.8	1.8	1.8	1.8
CD90 FITC	1.8	1.8	1.9	1.9	1.8
**Markers**	**Antibody concentration (μl)**				
	**1**	**2**	**5**	**10**	**20**
CD11c APC	1.6	1.4	1.3	1.4	1.4
CD19 APC	2.3	2.8	2.9	2.7	2.4

The immunophenotype validation required a first step which is the tritration of each antibody performed by using scalar antibody dilution. The best antibody concentration was that with a higher resolution index. The lowest antibody concentration was chosen when there was an equal resolution index.

As a second step, we performed Performance Qualification (PQ), as explained above, in compliance with ICHQ2 [2]. We evaluated the time trend for five consecutive days for each type of bead and we verified the stability over time of the cytometer set up (data not shown).

**Figure 5:**
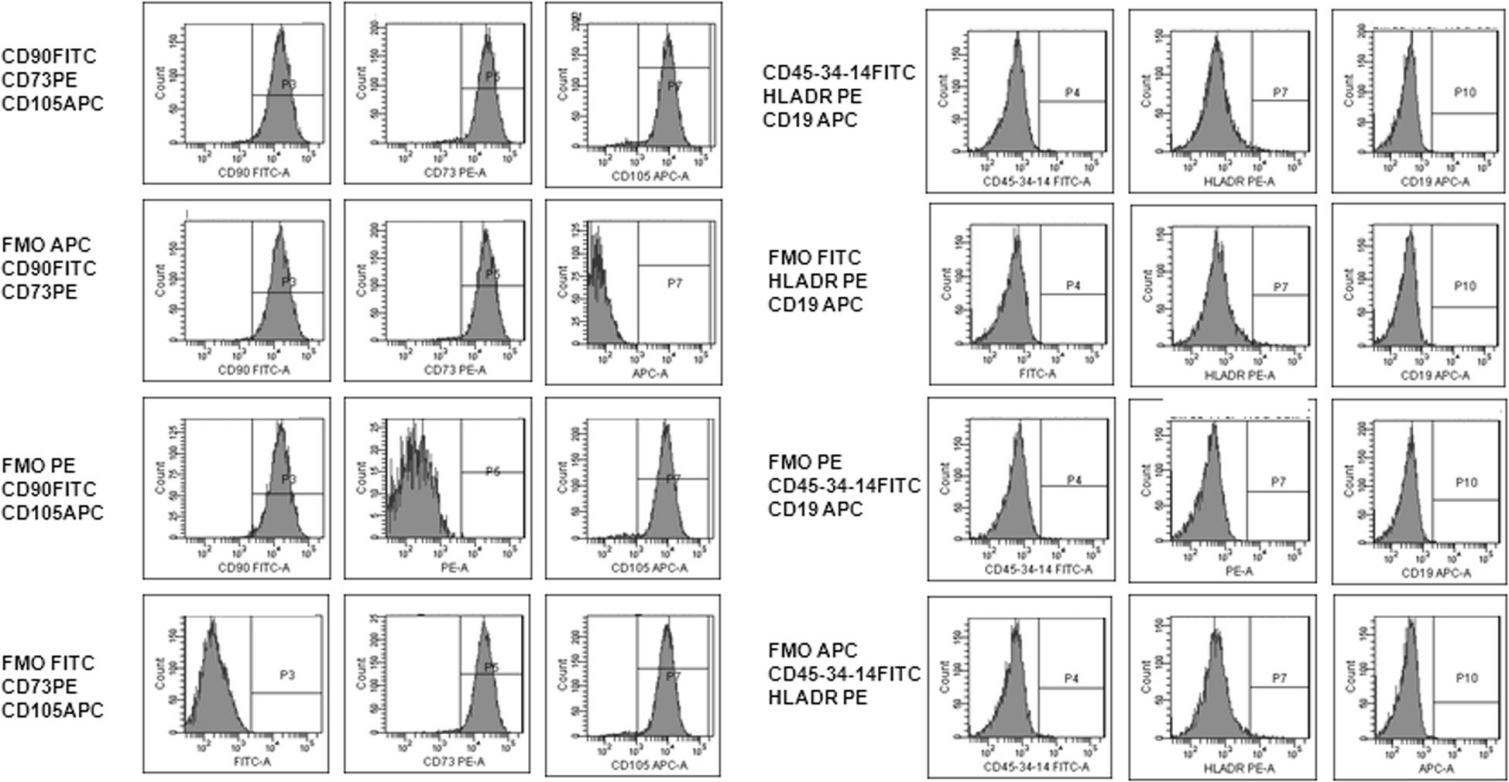
**Representative panel of fluorescence minus one method (FMO).** Cytofluormetric analysis of BM MSCs using FMO.

According to ICHQ2 [2] guidelines and the EU Pharmacopoeia [3] we evaluated specificity on BM MSCs, iDCs, mDCs and CTLs, as described above, and shown by representative panel of BM MSCs in Figure 5.

**Figure 6:**
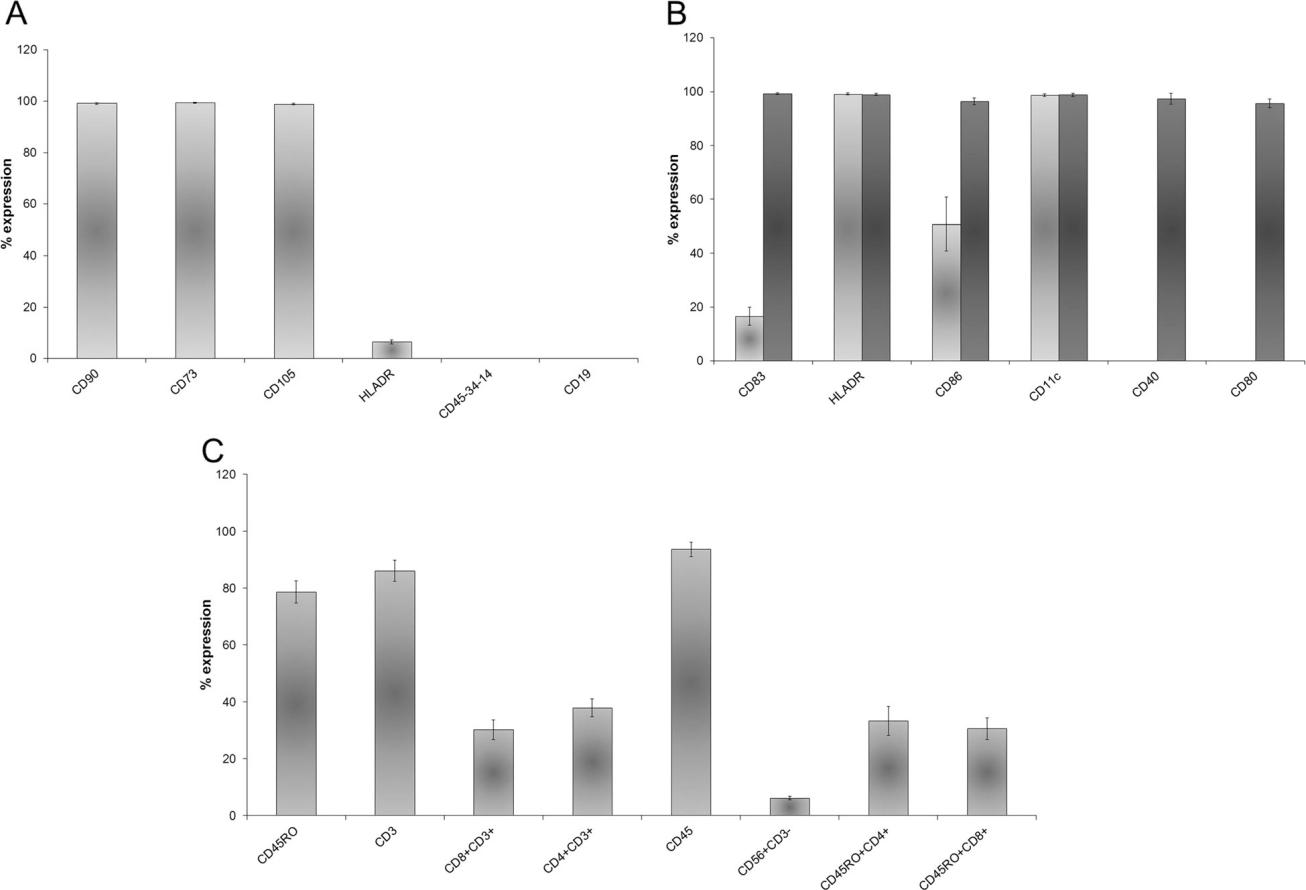
**Immunophenotype specificity.** The histograms show the mean percentage expression of three experiments of each marker for BM MSCs **(A)**, iDCs (B-light grey histograms), mDCs (B-dark grey histograms) **(B)** and CTLs **(C)**. Bars are SD.

BM MSCs were negative for CD45, CD14, CD34 hematopoietic surface antigens, CD19 B lymphocyte antigen and HLADR not expressed on mesenchymal stem cells in an unstimulated state. BM MSCs expressed high levels of CD90 (mean 99.2% ± 0.31), CD73 (mean 99.4% ± 0.27), CD105 (mean 98.9% ± 0.21) (Figure 6A).

DCs were selected from PBMCs using an adhesion method and cultured in specific medium with rhGM-CSF [11] and rhIL-4 [10]. After 5 days of culture iDCs expressed a low level of CD83 (mean 16.6% ± 3.31), CD86 (50.8% ± 9.95) and a high level of CD11c (mean 98.7% ± 0.50) and HLADR (mean 99.1% ± 0.41), but not CD40 and CD80 (Figure 6B). The mDCs expressed a high level of CD83 (mean 99.3% ± 0.30), CD86 (mean 96.3% ± 1.26), CD40 (mean 97.4% ± 2.06), CD80 (mean 95.7% ± 1.65), HLADR (mean 99% ± 0.40) and CD11c (mean 98.8% ± 0.56) (Figure 6B).

For anti-tumor CTLs induction, donor derived PBMCs were stimulated with mDCs pulsed with irradiated human osteosarcoma cell lines, used as the source of tumor Ag. CTLs were expanded in an Ag-independent way with rhIL-2 and OKT3.

After at least 7 days of Ag-independent expansion, we obtained the following populations: CD3+ (mean 86.2% ± 3.80), CD3 + CD8+ (mean 30.3% ± 3.43), CD3 + CD4+ (mean 38% ± 3.23), CD45RO + CD4+ (mean 33.4% ± 5.08), CD45RO + CD8+ (mean 30.7% ± 3.77), CD56 + CD3- (mean 6.2% ± 0.70). The lymphocyte population was negative for CD45RA and CD19 (Figure 6C).

As previously explained, the immunophenotype test was performed on our CTPs three times by the same operator. To obtain the *inter* experiment CV% QC manager calculated the mean and SD of the MFI of three replicates for each cell type (BM MSCs, CTLs, mDCs, iDCs) for each marker. For each marker, the *inter* experiment CV% was ≤ 10%. All the data are summarized in Table 4. These data demonstrated that the method is both valid and precise.

**Table 4:** Immunophenotype precision

**BM MSCs Markers**	**CV% Inter-experiment**
CD90 FITC	6.22
CD73 PE	0.95
CD105 APC	1.86
CD45-34-14 FITC	3.13
HLADR PE	4.46
CD19 APC	3.84
**iDCs Markers**	**CV% Inter-experiment**
CD83 FITC	2.2
CD40 PE	10
HLADR PERCPCY5	4.6
CD11c APC	2.7
CD80 FITC	8.4
CD86 PE	7.1
**mDCs Markers**	**CV% Inter-experiment**
CD83 FITC	3.6
CD40 PE	7.9
HLADR PERCPCY5	6.3
CD11c APC	5.4
CD80 FITC	5.1
CD86 PE	3.9
**CTL-LS Markers**	**CV% Inter-experiment**
CD3 FITC	9.7
CD8 PE	10
CD4 APC	8.1
CD45 PERCP	8.1
CD56 PE	7.0
CD19 APC	8.0
**CTL-E Markers**	**CV% Inter-experiment**
CD45RA FITC	9.4
CD45RO PE	10
CD4 APC	3.7
CD3 PERCP	6.9
CD8 APC	6.4

The immunophenotype test was performed three times under the same operating conditions by the same operator on our CTPs. The *inter* experiment CV% was calculated considering the mean and SD of the mean fluorescence intensity (MFI) of each cytofluorimetric marker.

The acceptance criteria was an inter-experiment CV% ≤ 10%.

*Abbreviations:CTL LS* Cytotoxic T Lymphocyte subpopulations, *CTL E* Cytotoxic T Lymphocyte effectors.

## Discussion

Cellular therapy is an emerging field in medicine. All the cell medicinal products must be produced in compliance with current GMP guidelines for medicinal products and investigational medicinal products for human use [7,18-24]. During CTP manufacturing, critical steps should be considered to demonstrate their suitability for routine processing and should be validated in order to produce cells of the required quality. All biological products must meet the prescribed requirements and no lot of any licensed product may be released by the manufacturer prior to the completion of tests for the conformity with standards applicable to such products [25]. In order to guarantee sterility, in accordance with international guidelines [7], one of the parameters that needs to be monitored in the manufacturing phases and in lot release is the endotoxin level. The LAL test is used to rule out that the products, given to patients, will cause toxic reactions, resulting from pyrogen contamination. On these bases, we have successfully validated, in compliance with the EU Pharmacopeia [3], endotoxin testing of BM MSCs and CTLs as cell therapy products.

By evaluating specificity and the detection limit in compliance with ICHQ2 [2], we demonstrated that the endotoxin chromogenic method, validated in accordance with the EU Pharmacopoeia [3], is suitable as a release test for our CTPs.

Although Soncin at al. [25] demonstrated the possible use of an alternative method for endotoxin evaluation in cell based products, for our purposes, we chose to validate the endotoxin test, a traditional method, that has been both widely used in the pharmaceutical industry and suggested by the EU Pharmacopeia.

For the batch release of CTPs used in clinical protocols, to satisfy pharmaceutical quality requirements [7] for cell identity determination, the immunophenotype is a fundamental parameter to be assessed.

On the basis of our previous pre-clinical papers on BM MSCs, DCs and CTLs reporting the characterization of the cell identity and to data published by other authors in this field [9,12,13,26,27], the aim of our work was simply to validate the analytical procedure of immunophenotyping, according to European Parmacopoeia [3] and ICHQ2 [2], on our CTPs and not to assay cell potency. Furthermore, we referred to the above described data, using cells prepared in the same way, as robust data to set up, in our Validation Master Plan, the acceptance criteria of the identity of every cell type analysed.

According to ICHQ2 for immunophenotyping, which is an identity assay, we tested specificity by FMO. In the present study we have demonstrated that the immunophenotype test is validated according to the current rules in the cell therapy field as it is able to discriminate the populations of interest.

The immunophenotype method for BM MSCs characterisation was considered specific as they expressed high level of CD90, CD73, CD105 and were negative for CD45, CD14, CD34, CD19 and HLADR and moreover they were able to adhere to the plastic in standard culture conditions and to differentiate into osteoblasts, adypocytes, and chondrocytes [8] (data not shown), in compliance with the International Society for Cellular Therapy (ISCT) guidelines, that specify the minimal criteria to define human MSCs [14].

We also analysed DCs, which are used as antigen presenting cells (APCs) [26-28] for the *in vitro* generation of tumor specific CTLs. The immunophenotyping was specific as iDC expressed a low level of CD83, CD86, a high level of CD11c, HLADR and were negative for CD40 and CD80. In contrast the mDCs, after a maturation step with a cytokine cocktail, showed the up-regulation of co-stimulatory molecules that are crucial in determining whether engaged T lymphocytes become anergic or develop productive immunity [29]. Furthermore, the fact that CD83, one of the best-known maturation markers for human dendritic cells, is strongly up-regulated together with co-stimulatory molecules such as CD80 and CD86 during DC maturation suggests it plays an important role in immune responses induction [30].

The protocol used to generate anti-tumor CTLs includes two rounds of tumor-specific stimulation followed by an Ag-independent expansion [13]. Flow cytometry analysis of CTLs was specific as they were positive for CD3+, CD3 + CD8+, CD3 + CD4+, negative for CD19 and expressed low level of CD56 + CD3-, according to our acceptance criteria, and in addition they were able to kill specific target (data not shown). Our data are in agreement with those which show that CD4+ T cells are also involved in anti–tumor effector activity through a perforin-mediated mechanism [15]. Their results supported the central role played by CD4+ T cells not only in providing help for optimal priming and expansion of anti-tumor CD8+ T cells, but also as active effectors of the immune response [31,32]. Furthermore, according to published data reporting that the expression of CD45 isoforms in human T cell distinguishes naïve T cells (CD45RA+) from memory (CD45RO+) T cells [33], the phenotypic analysis of our CTLs showed CD45RO + cells in both CD4 and CD8 subsets and were negative for CD45RA. Recent studies indicate that memory T Lymphocytes contain distinct populations of central memory (TCM) and Effector Memory (TEM) cells characterized by distinct homing capacity and effector function [34].

Although accuracy, repeatability or detection limits are not required for identity test validation, we did, however, decide, to verify precision of every CTPs by performing immunophenotype staining and analysis of only one sample in triplicate and work out the inter experiment CV%.

The LAL test is instead a limit test for the control of impurities and, for PQ assessment, specificity and detection limit validation are required under ICHQ2. Moreover, PE gives a good description of the LAL test in terms of accuracy, linearity, detection limit and specificity by seeding each sample in duplicate, using a standard curve and spike recovery, respectively. We followed these requirements to reach the task, carrying out the test in triplicate on the same samples.

Our validation policy in this context was due to the fact that the software used to this purpose is not CFR21 compliant [35-37]. So, in order to ensure our validation results, we decided to validate only the QC Manager performing tests in triplicate on the same samples. The future role of the QC Manager will be the training of the other Qualified Operators (QOps).

## Conclusions

In conclusion, according to ICH guidelines [2], this validation protocol showed that analytical methods for endotoxin and immunophenotype analysis may be used as quality controls for the batch release of CTPs, prepared in clean rooms and in GMP conditions, for clinical cell-based protocols.

Thanks to the data present in this study, together with those previously described by Gunetti et al. [5] we demonstrated the feasibility of the validation of analytical methods for cell therapy products; and thus our paper could offer an important contribution for the scientific community in the field of CTPs, above all to small Cell Factories such as ours, if it is not always possible to have CFR21 compliant software.

## Abbreviations

APC: Allophycocyanin; APCs: Antigen presenting cells; BM MSCs: Bone marrow mesenchymal stem cells; CC: Correlation coefficient; CTLs: Cytotoxic T lymphocytes; CTP: Advanced therapy products; CV%: Coefficient of variation percentage; DCs: Dendritic cells; EU: Endotoxin unit; EU Pharmacopoeia: (EU) Pharmacopoeia; FBS: Fetal bovine serum; FITC: Fluorescein isothiocyanate; FMO: Fluorescence minus one; GMP: Good manufacturing practice; GM-CSF: Granulocyte-macrophage colony-stimulating factor; HS: Human serum; ICH Q2: International conference on harmonization Q2; IL: Interleukin; IQ: Installation qualification; iDCs: Immature dendritic cells; LAL: Limulus amebocyte lysate; LPS: Lipopolysaccharide; mAb: Monoclonal antibodies; mDCs: Mature dendritic cells; MFI: Mean fluorescence intensity; OQ: Operational qualification; PBS: Phosphate buffer saline; PBMCs: Peripheral blood mononuclear cells; PE: Phycoerythrin; PerCP-Cy5.5: Peridinin-chlorophyll protein cyanine 5.5; PerCP: Peridinin-chlorophyll protein; PGE2: Prostaglandin E2; PPC: Positive product control; PQ: Performance qualification; QC: Quality control; QOps: Qualified operators; SD: Standard deviation; TNF: Tumor necrosis factor; VMP: Validation master plan; wBM: Whole bone marrow.

## Competing interest

The authors declare that they have no competing interests.

## Authors’ contributions

DR participated in the design of the study, carried out the experiment, acquired/ analyzed /interpreted data, performed the statistical analysis, and drafted the article. SC participated in the design of the study, analyzed/ interpreted data, and performed the statistical analysis. MG participated in the design of the study, interpreted data, and performed the statistical analysis. KM participated in the design of the study, interpreted data, and performed the statistical analysis. ES participated in the design of the study, carried out the cell biology studies, interpreted data, and performed the statistical analysis. MM participated in the design of the study, interpreted data, and performed the statistical analysis. LC participated in the design of the study, interpreted data, and performed the statistical analysis. FS participated in the design of the study, interpreted data, and performed the statistical analysis. ML participated in the design of the study, interpreted data, and performed the statistical analysis. IF conceived of the study, participated in the design of the study, interpreted data, drafted the article FF conceived of the study, contributed reagents/materials/analysis tools and interpreted data. All authors revised the article critically for important intellectual content, read and approved the final manuscript.

## Authors’ information

DR: MSc, Head, Quality Control.

SC: PhD, Qualified Operator, Production.

MG: PhD, Qualified Operator, Quality Control.

KM: BSc, Head, Production.

ES: PhD, Qualified Operator, Production.

MM: PhD, Qualified Operator, Production.

FS: DipBiol, , Qualified Operator, Production.

ML: DipBiol, , Qualified Operator, Quality Control.

LC: PhD, Qualified Operator, Quality Control.

IF: MSc, Qualified Person, Quality Assurance.

FF: MD, Director.
